# Predictors of Death among Patients Who Completed Tuberculosis Treatment: A Population-Based Cohort Study

**DOI:** 10.1371/journal.pone.0025315

**Published:** 2011-09-28

**Authors:** Juan-Pablo Millet, Angels Orcau, Cristina Rius, Marti Casals, Patricia Garcia de Olalla, Antonio Moreno, Jeanne L. Nelson, Joan A. Caylà, the Barcelona Tuberculosis Working Group

**Affiliations:** 1 Epidemiology Service, Public Health Agency of Barcelona, Barcelona, Spain; 2 CIBER de Epidemiología y Salud Pública, Madrid, Spain; 3 Departament de Pediatria, Ginecologia i Medicina Preventiva, Universitat Autònoma de Barcelona, Barcelona, Spain; 4 Departament de Salut Pública, Universitat de Barcelona, Barcelona, Spain; 5 Departament de Ciencies Basiques, Universitat Internacional de Catalunya, Barcelona, Spain; University of Minnesota, United States of America

## Abstract

**Background:**

Mortality among patients who complete tuberculosis (TB) treatment is still high among vulnerable populations. The objective of the study was to identify the probability of death and its predictive factors in a cohort of successfully treated TB patients.

**Methods:**

A population-based retrospective longitudinal study was performed in Barcelona, Spain. All patients who successfully completed TB treatment with culture-confirmation and available drug susceptibility testing between 1995–1997 were retrospectively followed-up until December 31, 2005 by the Barcelona TB Control Program. Socio-demographic, clinical, microbiological and treatment variables were examined. Mortality, TB Program and AIDS registries were reviewed. Kaplan-Meier and a Cox regression methods with time-dependent covariates were used for the survival analysis, calculating the *hazard ratio* (HR) with 95% confidence intervals (CI).

**Results:**

Among the 762 included patients, the median age was 36 years, 520 (68.2%) were male, 178 (23.4%) HIV-infected, and 208 (27.3%) were alcohol abusers. Of the 134 (17.6%) injecting drug users (IDU), 123 (91.8%) were HIV-infected. A total of 30 (3.9%) recurrences and 173 deaths (22.7%) occurred (mortality rate: 3.4/100 person-years of follow-up). The predictors of death were: age between 41–60 years old (HR: 3.5; CI:2.1–5.7), age greater than 60 years (HR: 14.6; CI:8.9–24), alcohol abuse (HR: 1.7; CI:1.2–2.4) and HIV-infected IDU (HR: 7.9; CI:4.7–13.3).

**Conclusions:**

The mortality rate among TB patients who completed treatment is associated with vulnerable populations such as the elderly, alcohol abusers, and HIV-infected IDU. We therefore need to fight against poverty, and promote and develop interventions and social policies directed towards these populations to improve their survival.

## Introduction

Tuberculosis (TB) continues to be one of the leading causes of death from infectious diseases worldwide [1]–[2]. Its mortality especially affects low income countries with high incidence rates of human immunodeficiency virus (HIV) infection. However, in developed countries, where TB is less common and mortality has gradually declined in recent decades, TB patients also die, though not always from TB itself [3]–[7].

Various studies have reported TB mortality and associated factors during active disease [4]–[5], [7]–[9] and have emphasized the importance of treatment adherence to reduce the probability of dying [3], [7]–[11]. However, there is little data about the long-term survival for successfully treated TB patients. It has been suggested that the treatment outcome may not reflect final patient status, in part due to pulmonary impairment after TB disease [12]. This has implications for patient care, such as more aggressive case prevention strategies and post-treatment evaluation, as well as for TB control of default and lost to follow-up cases [12]–[14].

The HIV and injecting drug users (IDU) epidemics in developed countries during the 1990's had a considerable impact on TB rates and mortality [15], [16]. It is therefore of interest to determine the status of the TB patients who survived and whether they still represent a vulnerable group, despite drug abuse care programs and the use of highly active antiretroviral therapy (HAART).

The identification of the characteristics of patients who die prematurely will help identify the most vulnerable populations and help to target them in future public health interventions. Therefore, the aim of this study was to determine the incidence of death and its predictors in a cohort of successfully treated TB patients.

## Materials and Methods

### Ethics statement

Demographic and clinical data were obtained from the epidemiological questionnaire used by TB Prevention and Control Program (TBPCP). The data was treated and analysed anonymously. The analysis was carried out retrospectively and involved data collected on a routine basis within the National Tuberculosis Program approved by the Spanish Ministry of Health. Therefore, no ethical approval nor informed consent was required. All data were treated in a strictly confidential manner following the ethical principles of the Helsinki Declaration of 1964 revised by the World Medical Organization in Edinburgh, 2000 and the Organic Law 15/1999 of Data Protection in Spain.

### Setting

The study was conducted in Barcelona (Catalonia, Spain), which had a population of 1,508,805 inhabitants living in an area of 100.4 square km during the enrollment period. The TBPCP has been operating in the city since 1987.

### Study design and population

This retrospective population-based cohort study included all patients detected by the TBPCP who began treatment between October 1^st^, 1995 and October 31^st^, 1997, with culture confirmation and drug susceptibility testing (DST) results, and who resided in the city of Barcelona. Cases that completed treatment according to the European recommended treatment outcome definition [17] were selected and followed to determine mortality rates and factors associated with death. The follow-up period continued until December 31^st^, 2005, at which time all cases were classified as either survived, transferred, or dead.

### Definitions

A “definite TB case” was defined using international recommendations [14]: a patient was considered to have TB if the culture was positive for *M. tuberculosis* complex. All patients who completed TB treatment, regardless of the availability of bacteriological confirmation, were considered cured [8], [18]. TB recurrence was defined as any new clinical and/or microbiological TB diagnosis presented by a patient who completed treatment after being TB disease-free for at least one year since treatment completion [19]–[20].

### Variables and information sources

All data came from the Barcelona TBPCP epidemiological surveys preformed by public health nurses on all detected cases. The Epidemiology Service collects data on TB and AIDS cases reported by physicians and also performs active surveillance for undeclared cases (via microbiological laboratories, hospital discharges, the city mortality registry, and social services). Socio-demographic variables included age, sex, country of birth (foreign or Spain), city district residence (inner-city or other), homelessness, prison history, smoking, alcohol abuse and IDU. Consumption of over 280 g of alcohol per week for men and over 168 g for women was considered alcohol abuse. Clinical variables included presence of HIV infection and route of infection, recurrence, type of TB (pulmonary, extrapulmonary or both (mixed TB) and chest radiograph results (abnormal non-cavitary, normal, or caviatry). Microbiological and treatment variables included smear test results, type of resistance (none, primary or secondary), drug resistance to isoniazid and rifampin (multi-drug resistance, or MDR), previous TB treatment, and treatment under directly observed treatment (DOT). DOT was used for some patient groups and defined as a observation from a healthcare worker of a patient as they take their TB medication. Primary resistance was defined as the presence of resistance to one or more anti-TB drugs in strains obtained from patients who had never received treatment. Secondary resistance was defined as resistance to one or more anti-TB drugs in strains recovered from patients who had received previous anti-TB treatment.

After a patient was included in the study, they were actively followed to verify vital status at the end of the study, as well as any recurrent TB episode during the follow up and/or date of transfer-out. Hospital clinical records, primary health care registries, the municipal census, the city mortality registry, and the Barcelona city drug abuse information system were reviewed to minimize lost to follow-up patients and compare duplicated information. At the end of the study, patients were considered to be lost to follow-up if no vital status or moving date was available.

### Statistical analysis

A descriptive analysis of the cohort was preformed, which included the medians and interquartile range (IQR) for quantitative variables, because none of the quantitative variables displayed normal distribution. The χ^2^ test was used for categorical variables and two-sided Fisher test were used when applicable. The overall mortality rate was calculated, as well as that specific to immigrants, HIV-infected patients and IDU, expressed in terms of cases per 100 person-years of follow-up (PY) and relative risks (RR). Follow-up time to death, transfer out, or end of the study was calculated in reference to the time elapsed since the end of TB treatment.

Patients who died were compared to the rest of the cohort. In the bivariate analysis, survival curves were estimated using the Kaplan-Meier curves and were compared using the Log-rank test. Possible interactions between patient clinical and demographic characteristics were considered and a forward inclusion approach was used. On a multivariate level, a Cox proportional hazards regression was preformed with time dependent covariates in relation to TB recurrence. Variables significantly correlated at the univariate level (p-value<0.10), and those of epidemiological interest. *Hazard Ratios* (HR) with their 95% confidence intervals (CI) were used to measure association. The proportional hazards assumption was tested by graphical methods and by goodness-of-fit analysis using Schoenfeld residuals. Analyses were preformed using the statistical packages: SPSS, v. 13.0 (SPSS Inc., Chicago, IL, USA) and R, v. 2.6.0 (The R Foundation for Statistical Computing).

## Results

### Cohort selection

**Figure 1:**
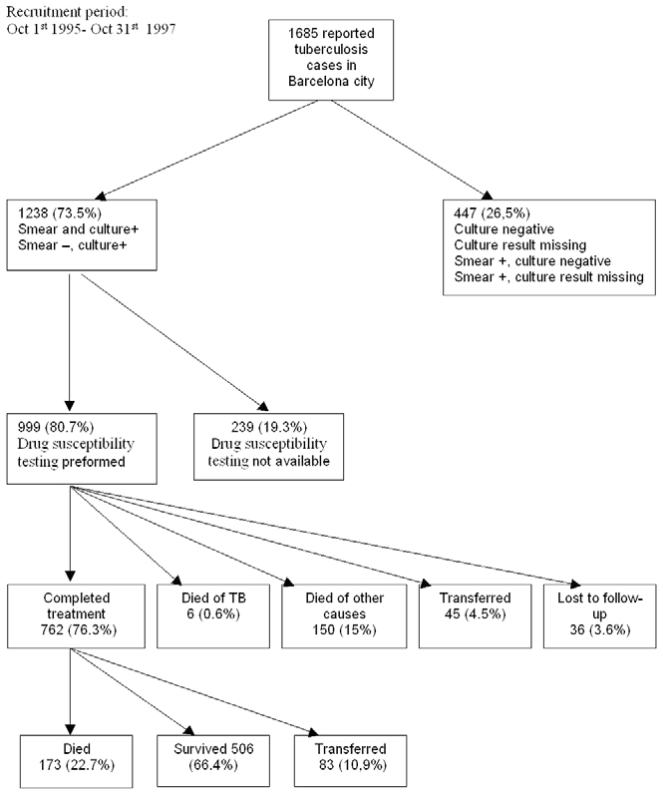
Flow chart of tuberculosis patient selection and evolution. Barcelona, 1995–2005.

Figure 1 shows the flow-chart of cohort selection. Of the 999 (80.7%) patients with culture *Mycobacterium tuberculosis* growth and DST results, 762 (76.3%) cases correctly completed treatment and thus constituted the study cohort, 6 (0.6%) died from causes attributed to TB, 150 (15%) died due to other causes before finalizing treatment, 45 (4.5%) moved outside of Catalonia, Spain during treatment, and 36 (3.6%) were lost to follow-up before completing treatment.

### Cohort description

The median age of the cohort was 36 years (IQR 28–52), with a predominance of men, Spanish patients, smokers and alcohol abusers. One in a five patients lived in the inner-city district. The most frequent presentation of TB was pulmonary (PTB), followed by extra-pulmonary TB. Smear test results were positive in 419 (55%) cases. A total of 35 (4.6%) patients presented with some primary drug resistance, while 13 (1.7%) presented with some secondary drug resistance. During the follow-up, there were 30 (3.9%) cases of recurrence; 26 (86.7%) in men and 4 (13.3%) in women. Table 1 shows socio-demographic, clinical, microbiological and treatment characteristics by vital status at the end of the follow-up.

**Table 1:** Distribution of characteristics of the cohort of 762 patients according to vital status at the end of follow-up. Barcelona, 1995–2005.

Variables	Totaln (%)762 (100)	Diedn (%)173 (22.7)	Survivedn (%)589 (77.3)	p-value
**Median age (years) (IQR)** *	36 (28–52)	56 (36–71.5)	34 (27–46)	<0.001
**Sex**				
. Male	520 (68.2)	134 (77.5)	386 (65.5)	0.002
. Female	242 (31.8)	39 (22.5)	203 (34.5)	
**Country of birth**				
. Foreign	72 (9.4)	6 (3.5)	66 (11.2)	0.001
. Spain	690 (90.6)	167 (96.5)	523 (88.8)	
**Residence in inner-city**				
. Yes	152 (19.9)	40 (23.1)	112 (19)	0.010
. No	610 (80.1)	133 (76.9)	477 (81)	
**Homelessness**				
. Yes	53 (7)	15 (8.7)	38 (6.5)	0.340
. No	709 (93)	158 (91.3)	551 (93.5)	
**Prison history**				
. Yes	69 (9.1)	20 (11.6)	49 (8.3)	0.370
. No	693 (90.9)	153 (88.4)	540 (91.7)	
**Smoker**				
. Yes	414 (54.3)	94 (54.3)	320 (54.3)	0.450
. No	331 (43.4)	73 (42.2)	258 (43.8)	
. Unknown	17 (2.2)	6 (3.5)	11 (1.9)	
**Alcohol abuse**				
. Yes	208 (27.3)	69 (39.9)	139 (23.6)	<0.001
. No	554 (72.7)	104 (60.1)	450 (76.4)	
**HIV-IDU** **				
. HIV Negative - Non-IDU	573 (75.2)	112 (64.7)	461 (78.3)	<0.001
. HIV Positive –IDU	123 (16.1)	51 (29.5)	72 (12.2)	
. HIV Positive - Non-IDU	55 (7.2)	10 (5.8)	45 (7.6)	
. HIV Negative –IDU	11 (1.4)	0 (0)	11 (1.9)	
**Recurrence**				
. Yes	30 (3.9)	6 (3.5)	24 (4.1)	0.810
. No	732 (96.1)	167 (96.5)	565 (95.9)	
**Type of tuberculosis**				
. Pulmonary	573 (75.2)	118 (68.2)	455 (77.2)	0.038
. Mixed	78 (10.2)	25 (14.5)	53 (9)	
. Extrapulmonary	111 (14.6)	30 (17.3)	81 (13.8)	
**Chest- X-Ray**				
. Abnormal non-cavitary	447 (58.7)	109 (63)	338 (57.4)	<0.001
. Cavitary	225 (29.5)	33 (19.1)	192 (32.6)	
. Normal	77 (10.1)	28 (16.2)	49 (8.3)	
. Unknown	13 (1.7)	3 (1.7)	10 (1.7)	
**Type of resistance**				
. No resistance	705 (92.5)	157 (90.8)	548 (93)	0.860
. Primary	35 (4.6)	8 (4.6)	27 (4.6)	
. Secondary	13 (1.7)	3 (1.7)	10 (1.7)	
. Unknown	9 (1.2)	5 (2.9)	4 (0.7)	
**MDR TB** ***				
. Yes	11 (1.4)	3 (1.7)	8 (1.4)	0.900
. No	747 (88.1)	168 (97.1)	579 (98.3)	
. Unknown	4 (0.5)	2 (1.2)	2 (0.3)	
**Previous TB treatment**				
. Yes	116 (15.2)	33 (19.1)	83 (14.1)	0.078
. No	618 (81.1)	130 (75.1)	488 (82.9)	
. Unknown	28 (3.7)	10 (5.8)	18 (3.1)	
**DOT** ****				
. No	623 (81.8)	123 (71.1)	500 (84.9)	
. Yes	139 (18.2)	50 (28.9)	89 (15.1)	<0.001

*IQR: Interquartile range.

**IDU: Injecting drug users.

***MDR TB: Multi-drug resistant tuberculosis.

****DOT: Directly observed treatment.

Regarding TB risk factors, 134 (17.6%) were IDU and 178 (23.4%) were infected with HIV. Of the 134 IDU cases, 123 (91.8%) were HIV-infected. Regarding HIV transmission, 123 (69.1%) were IDU, 30 (16.9%) were heterosexual non-IDU, 16 (8.9%) were homosexual non-IDU, and the route of HIV infection was unknown in 9 (5.1%) cases.

The median follow-up duration among patients who completed TB treatment was 8.04 years (IQR 5.8–8.8). At the end of follow-up, 173 (22.7%) patients had died, 83 (10.9%) had moved outside of Catalonia, Spain, and a total of 506 (66.4%) were alive. The patients who died were older, male, Spanish, residents in the inner-city, alcohol abusers and HIV-infected IDU. Table 1 compares the patients who died during follow-up with the survivors. None of the 11 non-HIV-infected IDU died during follow-up and were excluded from the multivariate analysis.

### Mortality rates

The mortality rate among the 762 patients who correctly completed treatment was 3.4/100 PY, or 3,355/100,000 PY of follow-up (1.6/100 PY among<41 years, 2.9/100 PY among 41–60 and 8.4/100 PY among>60 years and 9.3/100 PY among>64 years old patients). The rate among IDU and among HIV-infected patients was 6.5/100 PY (RR: 2.0 CI: 1.5–2.6) and 5.9/100 PY (RR: 1.8 CI: 1.4–2.3), respectively. Among immigrant and native populations, mortality was 1.5/100 PY (RR: 0.3 CI: 0.2–0.8) and 3.5/100 PY (RR: 2.9 CI: 1.3–6.3), respectively.

The median duration of follow-up after TB treatment completion and prior to death was 3.7 (IQR 1.5–6.3) years. The cumulative probabilities of dying at 1, 3, 6 and 9 years of follow-up were 4.5, 11.1, 17.7, and 25.9 percent, respectively. The incidence of recurrence was 0.5/100 PY, or 530 cases per 100,000 inhabitants; 1.1/100 PY among HIV-infected patients, and 0.4/100 PY among non-HIV-infected.

### Factors associated with death

**Figure 2:**
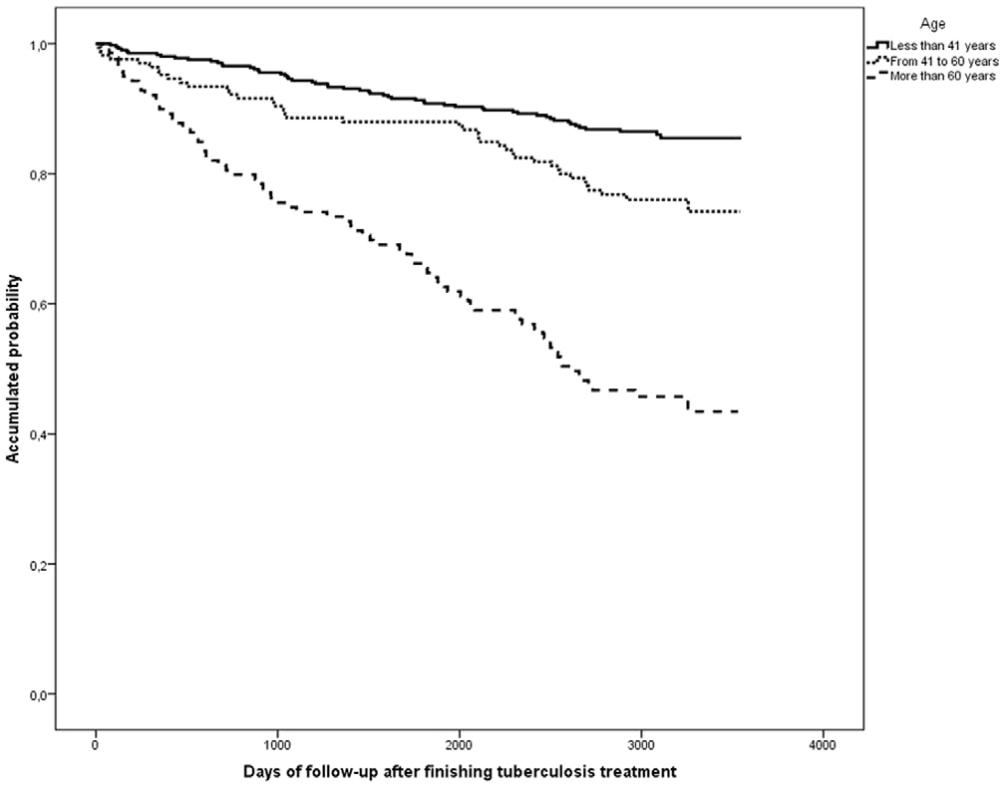
Kaplan Meier curves of the risk of death in a cohort of tuberculosis patients according to age group. Barcelona, 1995–2005.

**Figure 3:**
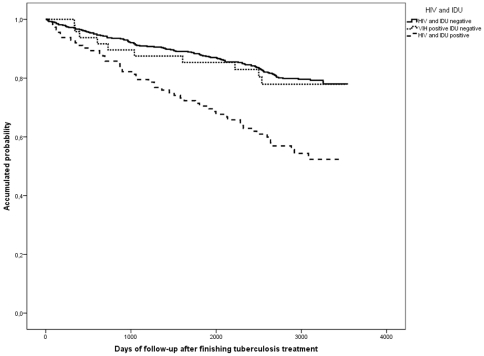
Kaplan Meier curves of the risk of death in a cohort of tuberculosis patients according to HIV infection and IDU status. Barcelona, 1995–2005.

**Figure 4:**
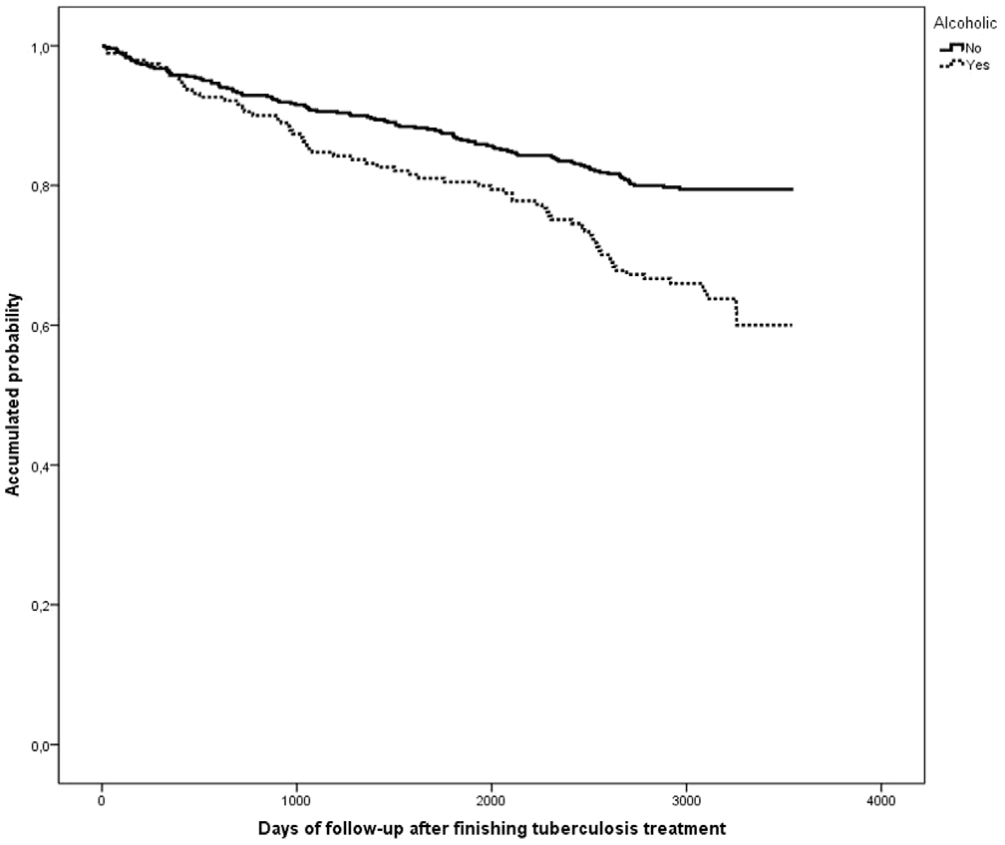
Kaplan Meier curves of the risk of death in a cohort of tuberculosis patients stratified according to presence of alcohol abuse. Barcelona, 1995–2005.

The following factors were significantly associated with death on a univariate level: male, age over 41 years, born in Spain, residence in the inner-city, alcohol, HIV-infected IDU, mixed TB, treatment under DOT, cavitary and normal chest-X-ray, and previous TB treatment (Table 2 and Figures 2, 3, 4). On the multivariate level, age was associated with death, with a significant gradient and 3.5-fold higher risk of dying after an age 41 years. HIV-infection and IDU were also significantly associated with a 7.9-fold higher risk of dying. Alcohol abusers had a 1.7 times higher risk. TB recurrence was not found to be significantly associated with death (Table 2).

**Table 2:** Factors associated with death in a cohort of 762 successfully treated tuberculosis patients. Barcelona, 1995–2005.

	Univariate Analysis			Multivariate Analysis		
Variables	Hazard Ratio	95%CI	p-value	Hazard Ratio	95%CI	p-value
**Age (years)**						
. Under 41	1	-	-	1	-	-
. 41 to 60	1.9	1.3–2.8	0.002	3.5	2.1–5.7	<0.001
. Older than 60	5.3	3.8–7.5	<0.001	14.6	8.9–24	<0.001
**Sex**						
. Female	1	-	-	1	-	-
. Male	1.7	1.2–2.4	0.005	1.1	0.8–1.7	0.795
**Country of origin**						
. Foreign	1	-	-	1	-	-
. Spain	2.4	1.1–5.5	0.030	1.5	0.7–3.5	0.315
**Residence in inner-city**						
. No	1	-	-	1	-	-
. Yes	1.4	1–2	0.060	1.2	1.8–1.7	0.335
**Alcohol abuse**						
. No	1	-	-	1	-	-
. Yes	1.9	1.4–2.6	<0.001	1.7	1.2–2.4	0.003
**HIV-IDU** *						
. HIV Neg- Non-IDU	1	-	-	1	-	-
. HIV and IDU	2.6	1.9–3.7	<0.001	7.7	4.5–13.3	<0.001
. HIV not IDU	1	0.5–2	0.953	1.4	0.7–2.8	0.350
**TB** ** **recurrence**						
. No	1	-	-	1	-	-
. Yes	1.6	0.7–3.7	0.237	0.8	0.3–2	0.563
**Chest- X-Ray**						
.Abnormal non-cavitary	1	-	-	1	-	-
. Cavitary	1.6	1.1–2.4	0.028	1.3	0.8–2.2	0.235
. Normal	0.6	0.4–0.9	0.008	0.7	0.5–1	0.068
. Unknown	0.9	0.3–2.9	0.890	0.7	0.2–2.2	0.521
**Previous TB treatment**						
. Yes	1	-	-	1	-	-
. No	1.6	1.1–2.3	0.020	1.3	0.9–2.1	0.185
. Unknown	2.2	1.2–4.2	0.015	1.1	0.6–2.4	0.719

*IDU: injecting drug users.

**TB: Tuberculosis.

## Discussion

Factors such as alcohol abuse, age, and being an HIV-infected IDU are associated with a higher risk of death, despite treatment completion for an episode of TB while TB recurrence or being non-IDU HIV infection were not associated. Several studies have analyzed the probability of dying during TB treatment [4]–[9], [20], but only few study patients who completed TB treatment to identify factors associated with the probability of dying [13], [14], [21], [22].

Almost a quarter of the study population died during the 8-year follow-up period. Mortality rate in our study population was higher (3,355 per 100,000 PY) compared to that of the general population (general mortality rate: 1,147 per 100,000 inhabitants) and mortality rate among older then 64 years was also higher (9,285 per 100,000 PY) compared to that of the general population>64 years old (4,843 per 100,000 PY) [23]. Higher mortality rate could be due to the considerable number of IDU, and high percentage HIV-infected IDU. Similarly, literature from the USA [12], [24], the Netherlands [21] and Finland [25] claim that TB patients have a higher risk of dying than the general population during TB treatment. However, it has also been suggested that the final treatment outcome may not reflect the patient's final status, perhaps in part due to pulmonary impairment after an episode of TB. This disease can produce changes and chronic lesions in the lungs such as bronchiectasis, pulmonary fibrosis that have been shown to be associated with decreased survival [12].

According to previous studies, the following factors were associated with death: age [5]–[8], [23], HIV infection [5], [8], [9], [14], [24], being IDU [8], [15], and alcohol abuse [5], [8], [24]. It has also been suggested that an increase in the risk of death may not be due to TB, but rather to individual factors such as HIV infection, alcohol or drug abuse, and precarious living conditions [8], [22], [25]. These modifiable risk factors provide a possibility of improvement through prevention and/or treatment; thus adherence to HAART among IDU patients should be warranted. Age, a non-modifiable factor, can also influence the final patient outcome. For example, older patients may experience longer diagnostic delays due to different clinical manifestations than younger patients [25]–[28]. The delay in diagnosing TB, probably due to the associated morbidity, as well as their lower immunity status in the older populations have been suggested as possible explanations for their increased mortality [28].

As expected in our setting [29], a considerable proportion of HIV-infected TB patients (23.4%) were IDU. Some studies have shown an increased mortality of up to 10 times higher in co-infected patients compared to non HIV-infected TB patients during the pre-HAART era. The increased use of HAART, which is effective and widespread in many high income countries but very often lacking in most rural areas in the low income countries, has led to changes in mortality among TB/HIV co-infected patients [30]. In Spain, HAART became available and free of cost in 1996. Unlike any other study [26], [31], we observed that co-infection with HIV among non-IDU is not associated with mortality. This could possibly be due to of better adherence to HAART than within the IDU population.

It is known that patients previously treated for TB have a higher risk of presenting MDR TB [31]–[33] and of dying [34] than new TB patients. However, in our study, previous TB treatment was not associated to mortality in the adjusted model. Abnormal non-cavitary and cavitary chest-X-ray were not associated to mortality in the adjusted model. Nevertheless, cavitary chest-X-ray showed a protection against mortality tendency at multivariate level (p-value 0.056) probably due to the well known association between good immune system and cavitary TB [35]. Type of TB was highly correlated with chest-X-Ray variable and was therefore excluded from the adjusted model.

Contrary to results of studies performed in the USA, the European Union, Mexico, or Azerbaijan [7]–[13], [36], [37], we did not find any association between MDR TB and mortality perhaps because our study population consisted of patients who completed TB treatment. Among the 156 excluded patients who died during treatment, only four of them (2.6%) were MDR-TB, 42 (26.9%) were IDU, and 59 (37.8%) were HIV-infected. Sixty-seven died in 1995–1996, when HAART treatment was still not as available. The prevalence of MDR-TB in our city is low because of the universal use of Fixed Dose Combination treatment and extensive DOT among vulnerable populations.

Despite existing evidence concerning the association of smoking with death and with TB [38], [39], we did not find a relationship between smoking and mortality (HR: 1.1 (CI 0.8–1.4), perhaps because the effects of tobacco occur over a longer period of time than our follow-up period of only eight years. We also found no effect of being foreign born on mortality, although some of the foreign born may have returned to their home countries and the final status was not recorded for some cases. Adjusting for type of TB instead of chest-X-Ray (correlated variables), disseminated TB presentation (mixed TB) was not associated with a higher risk of mortality as reported in a study from the USA [24]. As in similar settings [8], DOT was associated with a higher risk of death because it is usually implemented in the most vulnerable populations such as HIV infected, IDUs and alcohol abusers. Thus, it was considered as a confounder and not included in the multivariate analysis.

High TB recurrence rates have been reported in countries with high TB incidence and limited control programs [13], [22]. The recurrence rate found during the follow-up among our study population is one of the lowest when compared to those reported in recent publications [22], [40]–[43], ranging between 0–14%. Possible reasons for the recurrent rates in our study include lower TB incidence, smaller percentages of MDR TB, and/or varying inclusion criteria and definitions [40]. The existence of an effective TBPCP, which has prioritized the use of DOT strategy among high-risk groups to achieve compliance rates of 95% since 1995 [5], [8], could have also influenced our results. Additionally, few studies have assessed the influence of TB recurrence on mortality. We found that TB recurrence among patients who completed treatment was not associated with a higher risk of mortality. This was also observed in low incidence settings, such the USA [24], but not in high incidence settings such as Uzbekistan [13]. These differences could be due in part to the relapse and recurrence definitions. Above all, the lack of an association between TB recurrence and mortality in our study could be due to a high percentage (over 80%) of recurrent cases that correctly underwent treatment for their second TB episode.

Sputum samples frequently cannot be obtained from patients who complete treatment. For this reason and because the prevalence of drug-resistance is low, we considered treatment completion to be a good approximation for cured [13], [18]. Mortality data among HIV, alcohol and drug abusers in the general population was not available. Therefore, performing direct comparisons and calculations among these hidden populations is not possible. Other possible limitations of this population-based study include the limited access to clinical data such as HAART treatment and CD4 cell count, the limited presence of sub-populations who are usually culture negative such as children, and the absence of appropriate molecular techniques to determine if recurrence was due to reactivation of the same TB strain or due to re-infection by a different strain [44]. Identification of the origin of recurrence could have further implications in public health policy for TB [13], [31], [42]–[44]. Though the percent of lost to follow-up patients was small (3.6%), we compared lost to follow-up patients to the patients included in our analysis and found that the lost to follow-up population had a larger presence of foreign-born and inner-district residents. These patients could be underrepresented and could have perhaps skewed our final results in some way.

We conclude that HIV-infected IDU, those over 41 years old, and alcohol abusers have a poor long-term prognosis after completing TB treatment while TB recurrence and MDR TB are not associated with mortality when the first episode is successfully treated. The decrease in mortality among TB patients requires new public health interventions [44] and the enhancement of existing control programs to improve both prevention and treatment. Interventions should be directed at modifiable risk factors, such as alcohol abuse, treatment of HIV-infection and treatment of IDU patients. Priority must be given to promote relevant social policies to fight against poverty and achieve a decline of the mortality as well as an improvement in the quality of life and perceived health status of our patients.
